# Modulating effect of the PI3-kinase inhibitor LY294002 on cisplatin in human pancreatic cancer cells

**DOI:** 10.1186/1756-9966-27-76

**Published:** 2008-11-25

**Authors:** Masao Fujiwara, Kunihiko Izuishi, Takanori Sano, Mohammad Akram Hossain, Shoji Kimura, Tsutomu Masaki, Yasuyuki Suzuki

**Affiliations:** 1 Department of Gastroenterological Surgery, Faculty of Medicine, Kagawa University 1750-1, Miki, Kita, Kagawa 761-0793, Japan; 2 Department of Pharmacology, Faculty of Medicine, Kagawa University 1750-1, Miki, Kita, Kagawa 761-0793, Japan; 3 Department of Gastroenterology and Neurology, Faculty of Medicine, Kagawa University 1750-1, Miki, Kita, Kagawa 761-0793, Japan

## Abstract

**Background:**

Chemoresistance is a serious problem in pancreatic cancer, but the mechanism of resistance and strategies against the resistance have not been elucidated. We examined the potential of the phosphatidylinositol 3-kinase (PI3K)/Akt inhibitor LY294002 to enhance the anti-tumor effect of cisplatin and investigated the mechanism of chemoresistance in pancreatic cancer cells using a combination therapy of cisplatin and LY294002, both *in vitro *and *in vivo*.

**Methods:**

Cisplatin and LY294002, individually or in combination, were given to AsPC-1 and PANC-1 cell lines. Tumor growth, DNA fragments, and Akt phosphorylation were examined *in vitro*. To examine the therapeutic effect of cisplatin and LY294002, individually or combination an AsPC-1 tumor xenograft model was prepared for *in vivo *study.

**Results:**

Cisplatin induced growth inhibition and Akt phosphorylation in pancreatic cancer cells. LY294002 also inhibited cell proliferation but without showing Akt phosphorylation. However, the combination therapy markedly increased cleavage of caspase-3 and cytoplasmic histone-associated DNA fragments compared to the results with cisplatin alone. In the *in vivo *study, blocking the PI3K/Akt cascade with LY294002 increased the efficacy of cisplatin-induced inhibition of tumor growth in nude mice, suppressing half the tumor growth with cisplatin alone. There were no detectable side effects in mice treated with combination therapy.

**Conclusion:**

Our studies suggest that the PI3K/Akt pathway plays an important role in cisplatin resistance of pancreatic cancer cells. The augmentation of cisplatin with PI3K/Akt inhibitor may resolve the chemoresistance problem of cisplatin, and this might be a plausible strategy for achieving tolerance for chemotherapeutic agents in pancreatic cancer therapy.

## Background

Prognosis of pancreatic cancer is very poor. Forty percent of patients with pancreatic cancer show locally advanced disease, 40% shows metastatic disease, and 20% have resectable tumors. Even with radical surgery, the majority of patients recur [1,2]. Therefore, irrespective of resection, the key point for the treatment of pancreatic cancer is successful chemotherapy.

Currently, gemcitabine is regarded as the standard and only efficacious chemotherapeutic agent for advanced pancreatic cancer, even though it shows modest results and limited survival benefit [1-3]. When the first line of chemotherapy fails, there is no second effective chemotherapeutic agent for pancreatic cancer, because pancreatic cancer shows drug resistance to other single chemotherapeutics, such as platinum agents, topoisomerase inhibitors, and taxanes. This chemoresistance is a major therapeutic hurdle for patients with pancreatic cancer [4]. If a way can be found to overcome drug resistance, that would have wide use in cases the chemotherapy for pancreatic cancer. However, the molecular mechanism of chemoresistance in pancreatic cancer has not been clarified, though many theories have been discussed, such as loss of p53 function, increased DNA adduct repair, and overexpression of HER-2/new [5]. The phosphatidylinositol 3-kinase (PI3K)/Akt pathway has also been said one of the candidates for clarifying the chemoresistance of cisplatin [5]. PI3K/Akt pathway plays an important role in cell survival when cells are exposed to stimuli, such as withdrawal of growth factor, ultraviolet radiation, matrix detachment, cell cycle discordance, and DNA damage [6]. Akt is overexpressed in pancreatic cancer cells [7]. Therefore, the PI3K/Akt pathway might be an important survival pathway in the resistance of chemotherapy in patients with pancreatic cancer.

In establishing this theory, several studies have indicated that LY294002, a signaling inhibitor of the PI3K/Akt pathway, can modulate sensitivity to cancer chemotherapy *in vitro*. Brognard et al. [8] has demonstrated that Akt activity might promote therapeutic resistance in human non-small cell lung cancer cells and that LY294002 greatly potentiated chemotherapy-induced apoptosis in cells with high Akt levels, but not in cells with low Akt levels. Especially, Akt-mediated chemoresistance has been reported widely in ovarian cancer cases [9-13]. Moreover, LY294002 addition increased sensitivity of radiation and inhibited clonogenic growth [14]. All of these findings suggest the possibility of treating human malignancies using the PI3K/Akt inhibitor LY294002.

In the present study, we examined the molecular mechanism of resistance in chemotherapy. We choose cisplatin as a representative chemotherapeutic agent because it shows strong resistance for pancreatic cancer therapy, although it has been widely used for the treatment of many other cancer cases. We examined the effects of LY294002 *in vitro*, especially targeting the induction of apoptosis, as well as altered expression of phosphorylated Akt. We also evaluated the biological effects of this agent *in vivo *with mouse xenografts, testing the possibility that LY294002 might be useful as an antitumor drug for use in human pancreatic cancer.

## Methods

### Cell Culture and Cell Proliferation Assay

Human pancreatic cancer cell lines AsPC-1 and PANC-1 were used in this study. AsPC-1 and PANC-1 cells were seeded at a density of 2 × 10^3 ^cells/well on 96-well culture plates with complete culture medium and allowed to adhere to the plate overnight, and then the cells were incubated in the presence of LY294002 and/or cisplatin at various concentrations for 48 hours. Cell numbers were determined by the absorbance of each well at 490 nm using a MTS [3-(4,5-dimethylthiazol-2-yl)-5-(3-carboxymethoxyphenyl)-2-(4-sulfophenyl)-2H-tetrazolium] assay kit (CellTiter 96^® ^Aqueous One Solution Reagent (Promega, Madison, WI)) to examine the antitumor effect of cisplatin and/or LY294002.

### Immunoblotting

Cell lysates were prepared 6 hours after the treatment. Protein lysates were loaded (10 μg) and fractionated by 10% polyacrylamide gel. Specific bands were then visualized by western blotting using antibodies recognizing phosphorylated-Akt (Ser473), phosphorylated-Bad (Ser112), cleaved caspase-3, and β-actin. Horseradish peroxidase-conjugated goat antirabbit IgG was used as a secondary antibody for enhanced chemiluminescence.

### DNA Fragmentation Assay

We examined DNA fragmentation to assess apoptosis in AsPC-1 and PANC-1 cells. Cells were plated in 96-well plates 24 hours before treatment. DNA fragmentation was evaluated by examination of cytoplasmic histone-associated DNA fragments (mononucleosomes and oligonucleosomes) 48 hours after treatment with cisplatin and/or LY294002 using a Cell Death Detection ELISA^Plus ^kit (Roche Molecular Biochemical, Indianapolis, IN) according to the manufacturer's instructions.

### The *In Vivo *Experiment

BALB/C nu/nu 6-wk-old male mice purchased from Japan SLC, Inc., were used in this study. All mice were maintained under specific pathogen-free conditions at the Center for Animal Experimentation, Kagawa University, in accordance with the Guide for Animal Experimentation, Kagawa University. Mice received s.c. injection of 1 × 10^6 ^AsPC-1 cells in the right flanks. Seven days after injection, mice were randomized into four groups (n = 7 mice/group) to receive either vehicle (control), cisplatin (5 mg/kg), LY294002 (25 mg/kg), or cisplatin plus LY294002 intraperitoneally (i.p.). Cisplatin was injected once/week (total 3 times), and LY294002 was administered twice/week (total 6 times)). Mice were sacrificed on day 28 after tumor cell injections. Tumor growth was assessed weekly by measuring the two greatest perpendicular tumor dimensions. Tumor volume was calculated as follows: tumor volume (mm^3^) = [tumor length (mm) × tumor width (mm)^2^]/2 [15]. We measured the body weights and serum albumin, total bilirubin, and creatinine levels to examine the side effects.

### Statistical Analyses

Multiple one-way analyses of variance (ANOVA) followed by Scheffé's *post hoc *tests were used to assess differences among groups. Results were considered significant for *P *< 0.05. Data were expressed as the mean ± SD of the mean.

## Results

### Growth inhibition of pancreatic cancer cells by cisplatin or LY294002

**Figure 1:**
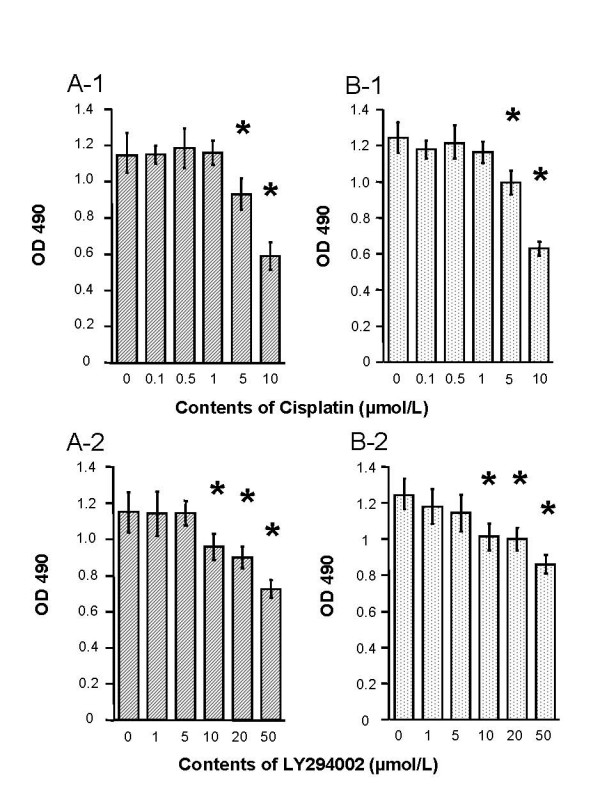
Growth inhibition of pancreatic cancer cells by cisplatin and/or LY294002. The antitumor growth effect of each single agent as well as the combination of agents was examined by MTS assay in 96-well plates at 2 × 10^3 ^cells/well. AsPC-1 and PANC-1 cells were cultured with cisplatin (0.1, 0.5, 1, 5, and 10 μmol/L) or LY294002 (1, 5, 10, 20, and 50 μmol/L) for 48 hours in DMEM-10% FBS. AsPC-1 cells were treated with cisplatin (A-1) and LY294002 (A-2). PANC-1 cells were treated with cisplatin (B-1) and LY294002 (B-2). A dose-dependent effect of cisplatin and LY294002 can be seen in these diagrams. Values represent the means (± SD) of n = 5. *P *< 0.05 compared with the controls.

Overall cell growth of AsPC-1 and PANC-1 pancreatic cancer cells treated with cisplatin or LY294002 at different concentrations ranging from 0.1 to 10 μmol/L and 1 to 50 μmol/L, respectively, were determined by MTS assay. As shown in Figure 1, both agents inhibited cell growth in a dose-dependent manner, and significant growth inhibition was demonstrated at the concentration of cisplatin 10 μmol/L and LY294002 50 μmol/L, 48 hours after treatment.

### Akt activation caused by cisplatin stimulation

**Figure 2:**
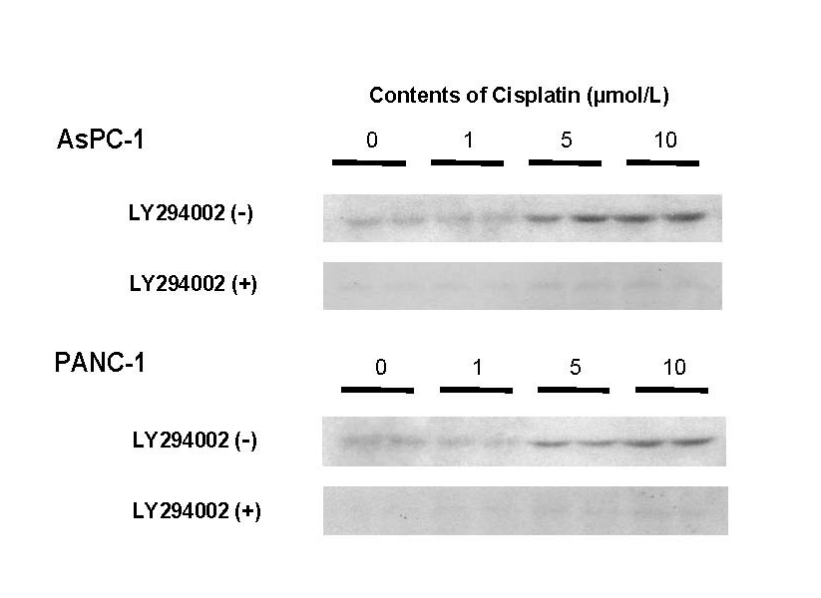
Akt activation caused by cisplatin stimulation. Western blotting shows the status of Akt activation using phospho-Akt antibody. Akt was phosphorylated at cisplatin concentrations over 5 μmol/L in both cell lines 6 hours after treatment. This phosphorylation was blocked by the addition of 10 μmol/L of LY294002.

Western blotting (Figure 2) shows the status of Akt activation using phospho-Akt antibody. Akt was phosphorylated over the concentration 5 μmol/L of cisplatin in both cell lines 6 hours after treatment. This phosphorylation was canceled by the addition of 10 μmol/L of LY294002.

### PI3 kinase inhibitor enhances the growth inhibitory effects of cisplatin

**Figure 3:**
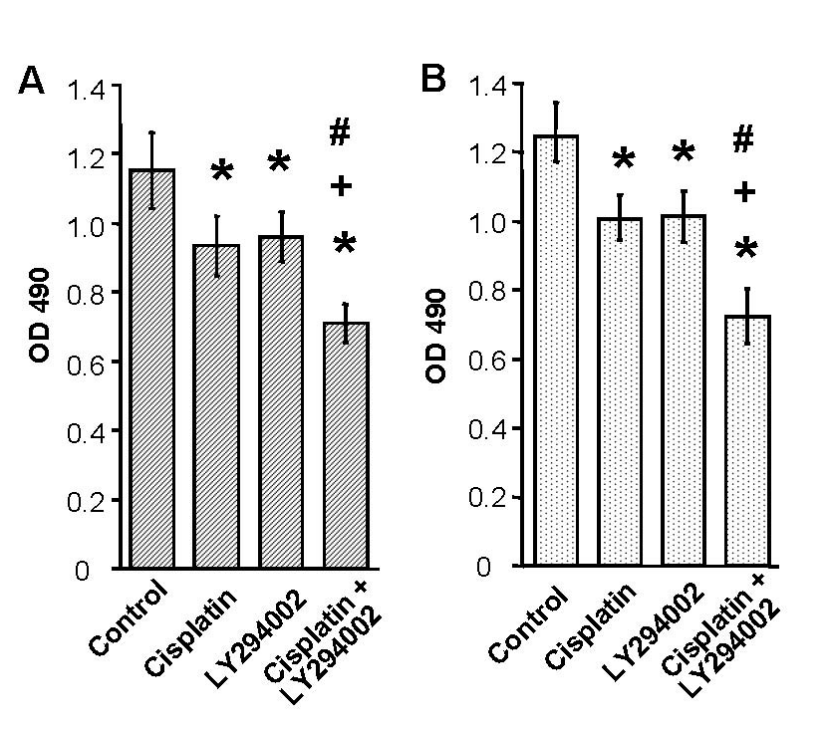
PI3-kinase inhibitor enhances the growth inhibitory effects of cisplatin. Cell proliferation was measured by MTS assay. The combined administration of 10 μmol/L LY294002 and 5 μmol/L cisplatin inhibited cell growth in AsPC-1 (A) and PANC-1 (B) significantly. Values represent the means (± SD) of n = 5. *, + and # compared with control, cisplatin treatment and LY294002 treatment group, respectively (*P *< 0.05).

To evaluate the effect of LY294002 on cisplatin treatment in pancreatic cancer cells, we evaluated cell proliferation using the MTS assay. In this experiment we selected a dose of 10 μmol/L of LY294002, which inhibited Akt phosphorylation caused by the stimulation of cisplatin in cell survival as shown in Figure 2. Augmentation of cisplatin with LY294002 inhibited cell growth in both cell lines (Figure 3). Compared with treatment with cisplatin or LY294002 alone, the combination treatment inhibited cell proliferation and viability more significantly (*P *< 0.05), 81%, 82%, 59% in AsPC-1 cells, and 81%, 83% and 61% in PANC-1 cells compared from control, respectively.

### Augmentation therapy with LY294002 enhanced the apoptotic pathway

**Figure 4:**
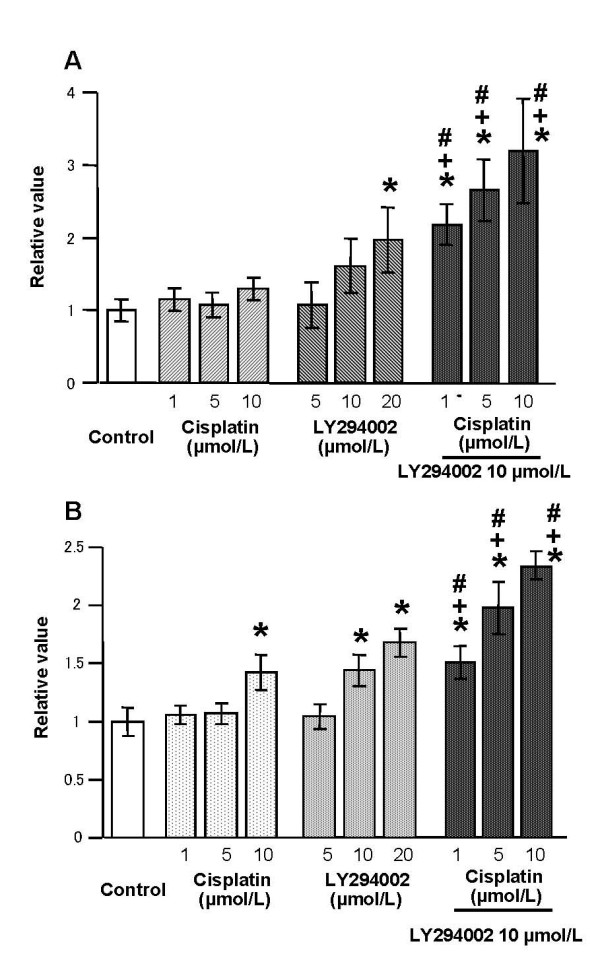
Augmentation therapy of LY294002 enhanced the apoptotic pathway in pancreatic cancer. Cytoplasmic histone-associated DNA fragments were determined by ELISA-based quantification. The pathway was increased significantly 48 hours after LY294002 administration, but apoptosis was not prominent following cisplatin treatment in AsPC-1 cell lines (A). The addition of 10 μmol/L LY294002 increased apoptosis in the presence of cisplatin synergistically. PANC-1 cell lines showed the same results (B). Values represent the means (± SD) of n = 5. *, + and # compared with control, cisplatin treatment and LY294002 treatment group, respectively (*P *< 0.05).

Apoptosis is an important mechanism for antiproliferative activity of many naturally occurring agents as well as synthetic agents. Apoptosis-induced treatment was assessed by ELISA-based quantification of cytoplasmic histone-associated DNA fragments. Treatment for 48 hours in AsPC-1 and PANC-1 cells with LY294002 resulted significant increase in the levels of cytoplasmic histone-associated DNA fragments at the dose of 20 μmol/L when compared with the control. This increase was not prominent in either cell line after receiving cisplatin treatment only (Figure 4A and 4B).

To examine the synergetic effect of cisplatin and LY294002, we evaluated cell growth at doses of 1, 5, and 10 μmol/L of cisplatin under the presence of 10 μmol/L LY294002, since the concentration was sufficient to inhibit Akt activation. Interestingly, the augmentation effect of LY294002 (at this concentration, LY294002 alone did not show significant apoptosis) in this apoptotic assay was more significant (Figure 4) than cell growth using the MTS assay (Figure 3) with 1.5 to 3 times increased levels of apoptosis assessed by DNA fragmentation in AsPC-1 and PANC-1 cells.

### Augmentation therapy modulates downstream effecters of Akt to apoptosis

**Figure 5:**
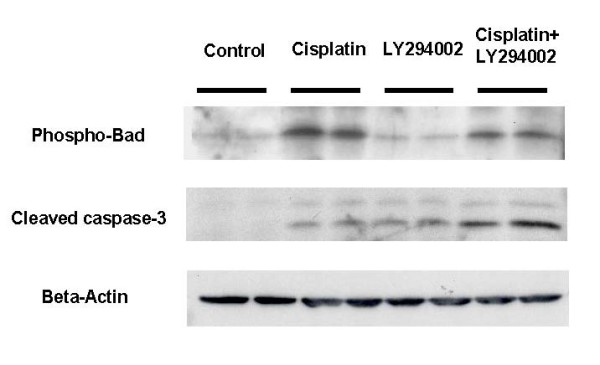
**Augmentation therapy modulates downstream effecters of Akt to apoptosis**. The downstream effects of the Akt pathway were examined by Western blotting. Bad was phosphorylated by cisplatin (5 μmol/L) treatment, while LY294002 (10 μmol/L) inhibited the phosphorylation. Augmentation with LY294002 weakened Bad phosphorylation and increased the cleavage of caspases-3.

There are multiple targets of the PI3K/Akt pathway that may mediate the ability of this cascade to promote cell survival during chemotherapy. Bad is a representative protein involved in cell death that has been shown to be regulated by Akt activity [13]. Upon activation of Akt, Bad is phosphorylated, resulting in its inactivation and the promotion of cell survival. As shown in Figure 5, phosphorylated levels of Bad protein are only slightly increased after the administration of cisplatin. However, combination therapy resulted in a lower phosphorylation level. In addition, combination therapy exacerbated the cleavage of caspase-3, an effecter of apoptosis, to a greater degree than did a single treatment of cisplatin.

### Synergetic effect of LY294002 on cisplatin therapy using mouse xenografts

**Figure 6:**
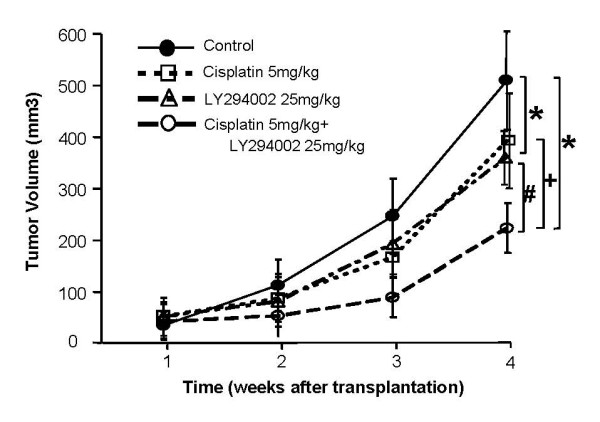
**Synergetic effect of LY294002 on cisplatin therapy using mouse xenografts**. AsPC-1 xenograft nude mice were treated with cisplatin and/or LY294002 for 3 weeks. Tumor volume in the mice administrated (i.p.) with cisplatin or LY294002 was slowly inhibited compared with the controls. Combination therapy showed significant inhibition compared to the results of single-agent treatments. Values represent the means (± SD) of n = 7. *, + and # compared with control, cisplatin treatment and LY294002 treatment group, respectively (*P *< 0.05).

A nude mouse model of pancreatic cancer was used to assess the *in vivo *synergetic effect of LY294002. Cisplatin at a dose of 5 mg/kg per week and LY294002 at a dose of 25 mg/kg twice/week were administered to mice bearing established AsPC-1 tumors for 3 weeks. As shown in Figure 6, at day 21, the control group of mice showed an increased tumor volume, while the group treated with cisplatin or LY294002 showed a decreased tumor volume by 77% and 70% compared to the controls, respectively. In addition, the combination treatment seemed to be more effective than the respective single treatments. The growth of tumor volume decreased to 44% of the volume in the controls, which was a significant difference, (*P *< 0.05).

### Side effects were not detected in combination therapy

To examine the side effect of combination therapy, the body weights and serum albumin, total bilirubin, and creatinine levels were examined at the end of therapy on day 28. No significant side effects were detected except for a slight elevation of ALT level.

## Discussion

All chemotherapeutic agents have inhibitory effect on tumor growth in high doses *in vitro*, as shown in Figure 1A-1 and 1B-1, however, the most important factor for successful chemotherapy *in vivo *is to think the safety range between therapeutic and toxic doses. Therefore, the up-regulation of chemosensitivity for cancer cells is important for chemotherapy without toxic effects. The chemosensitivity is defined by the intrinsic resistance which originally exists before chemotherapy, and acquired resistance which occurs during the period of chemotherapy. On the other hand, cisplatin has revealed itself as one of the most potent antitumor agents and has been applied to various human malignancies, such as cancers of the esophagus, head, neck, and ovary in the last 30 years, but cisplatin has not been found to be effective against pancreatic cancer [5]. Antitumor effect of cisplatin is based on the inhibition of DNA synthesis and RNA transcription, cell cycle arrest, and the induction of apoptotic pathways [5]. If we can up-regulate the sensitivity of cisplatin to pancreatic cancer cells, we will have a new therapeutic option for pancreatic cancer. Therefore, we examined the mechanism of the intrinsic resistance for cisplatin in pancreatic cancer cells. We found that (a) pancreatic cancer cells activated the PI3K/Akt pathway against cisplatin treatment; (b) inhibition of the PI3K/Akt pathway enhanced the antitumor effect of cisplatin, and (c) cisplatin induced apoptosis with subsequent deactivation of proapoptotic factors such as Bad and Caspace-3. Finally, we demonstrated that a combination therapy of cisplatin and LY294002 enhanced the antitumor effect in an *in vivo *model.

The PI3K/Akt pathway is one of the core intracellular signaling pathways in the stimulation of growth factors. Akt expression or its phosphorylated form has been reported as a significant prognosticator in sarcoma [16], gastric cancer [17], pancreatic cancer [18], and breast cancer [19]. Therefore, inactivation of the PI3K/Akt pathway should be effective as a specific chemotherapy against malignant tumors [20,21] because of lower expression of Akt in the surrounding normal tissue, and this type of chemotherapy should not have strong side effects. In addition, Akt plays an essential role as a survival signal pathway when cancer cells are exposed to cellular stress such as heat shock, oxidative stress, UV irradiation, matrix detachment, cell cycle discordance, DNA damage, and antitumor drug administration [22]. Therefore, DNA damage or reactive oxygen species induced by cisplatin may contribute to cisplatin resistance through the Akt activation [5].

Our data showed that Akt was up-regulated by cisplatin stimulation to prevent apoptosis. Therefore, inhibition of PI3K/Akt is a rational strategy against cancer cells to escape from cell death by cisplatin therapy. Several downstream effectors have been reported in PI3K/Akt phosphorylation-mediated cell survival. Bad, which is a pro-apoptotic Bcl-2 family member, can mediate cell survival by Akt phosphorylation [13]. Phosphorylated Akt can directly phosphorylate Bad, and may render Bad incapable of binding to Bcl-XL and restore the antiapoptotic function of Bcl-2, and regulate the activation of caspases through control of cytochrome c release from the mitochondria [23]. Our data also supported this pathway as a possible mechanism of cisplatin resistance. This form of cell death and apoptosis is a complex and well-organized process. The cytotoxicity of cisplatin activates many other signal transduction pathways, such as Fas/FasR, ATR, p53, and MAPK, and culminates in the activation of apoptosis through caspace-3 activation [5,24,25]. On the other hand, many biological defense systems are activated against this apoptotic stimulation, such as overexpression of HER-2/neu, loss of p53 function, and overexpression of antiapoptotic bcl-2 [5,26].

Many studies have been documented that LY294002 has a strong antitumor activity by inhibiting the PI3K/Akt pathway. However the majority of these studies were *in vitro *studies. The most important finding in the present study was in the *in vivo *experiment of pancreatic cancer therapy using cisplatin and LY294002. Also we examined the well tolerated toxicity of LY294002 because high dose of PI3K/Akt inhibitors interfere with the survival and/or proliferation of critical populations of normal cells and would display unacceptable toxicity when the PI3K/Akt pathway plays a critical role in many aspects of normal cellular homeostasis. Hu et al [27]. reported that daily i.p. administration of LY294002 at a dose of 100 mg/kg caused body weight loss and dry scaly skin in mice with ovarian cancer, presumably as a result of increased epidermal cell apoptosis that resulted in hyperkeratosis. Therefore, in the following study, they decreased shots of LY294002 administration to 3 days per week in combination with pacritaxel in ovarian cancer xenografts model within acceptable side effects [13]. Our results also showed similar result that twice a week i.p. administration of 25 mg/kg LY294002 was well tolerated.

In conclusion, the present study show that the combination of cisplatin and LY294002 has an additive or synergistic effect both *in vivo *and *in vitro *against human xenografts, i.e., an antitumor activity greater than that of each drug used as a single agent at the maximum tolerated dose. Our result presents a rational combination therapy based on the up-regulation of sensitivity for cisplatin and suggests a new strategy, the sensitization of pancreatic cancer to cisplatin, and provides a new application of an anticancer drug that has been considered non-effective.

## Competing interests

The authors declare that they have no competing interests.

## Authors' contributions

All the authors contributed as mentioned. MF and KI carried out *in vitro *experiment. KI, MAH and SK carried out *in vivo *experiment. TS performed western blotting of phospho-Akt. TM gave significant suggestion to this work and YS acts as moderator of this work.

**Table 1:** Side effects in combination therapy at the point of sacrifice.

	Control	Cisplatin (5 mg/kg)	LY294002 (25 mg/kg)	Cisplatin (5 mg/kg) + LY294002 (25 mg/kg)
Body weight (g)	23.7 ± 0.8	23.6 ± 0.5	23.8 ± 0.7	23.7 ± 0.8
Serum albumin (g/dl)	3.2 ± 0.2	3.1 ± 0.2	3.1 ± 0.3	3.2 ± 0.2
Total bilirubin (mg/dl)	0.04 ± 0.02	0.05 ± 0.01	0.06 ± 0.01	0.04 ± 0.02
Creatinine (mg/dl)	0.05 ± 0.02	0.06 ± 0.02	0.08 ± 0.02	0.05 ± 0.03
ALT (IU/l)	34.1 ± 9.7	31.5 ± 5.6	30.0 ± 6.7	44.1 ± 9.7 *

This table shows the body weights and serum albumin, total bilirubin, and creatinine levels at the end of therapy on day 28 *in vivo *experiments. No significant side effects were detected except for a slight elevation of ALT level in the combination therapy group. Values represent the means (± SD) of n = 7. *P *< 0.05 compared with LY294002 treatment group.
